# Telocytes in exercise‐induced cardiac growth

**DOI:** 10.1111/jcmm.12815

**Published:** 2016-03-14

**Authors:** Junjie Xiao, Ping Chen, Yi Qu, Pujiao Yu, Jianhua Yao, Hongbao Wang, Siyi Fu, Yihua Bei, Yan Chen, Lin Che, Jiahong Xu

**Affiliations:** ^1^ Regeneration and Ageing LabExperimental Center of Life SciencesSchool of Life ScienceShanghai UniversityShanghaiChina; ^2^ Department of GeriatricsXuhui Central HospitalShanghai Clinical CenterChinese Academy of ScienceShanghaiChina; ^3^ Department of CardiologyTongji HospitalTongji University School of MedicineShanghaiChina; ^4^ Department of CardiologyShanghai Yangpu District HospitalTongji University School of MedicineShanghaiChina

**Keywords:** telocytes, heart, exercise, CD34, vimentin, PDGFR‐α, β

## Abstract

Exercise can induce physiological cardiac growth, which is featured by enlarged cardiomyocyte cell size and formation of new cardiomyocytes. Telocytes (TCs) are a recently identified distinct interstitial cell type, existing in many tissues and organs including heart. TCs have been shown to form a tandem with cardiac stem/progenitor cells in cardiac stem cell niches, participating in cardiac regeneration and repair. Although exercise‐induced cardiac growth has been confirmed as an important way to promote cardiac regeneration and repair, the response of cardiac TCs to exercise is still unclear. In this study, 4 weeks of swimming training was used to induce robust healthy cardiac growth. Exercise can induce an increase in cardiomyocyte cell size and formation of new cardiomyocytes as determined by Wheat Germ Lectin and EdU staining respectively. TCs were identified by three immunofluorescence stainings including double labelling for CD34/vimentin, CD34/platelet‐derived growth factor (PDGF) receptor‐α and CD34/PDGF receptor‐β. We found that cardiac TCs were significantly increased in exercised heart, suggesting that TCs might help control the activity of cardiac stem/progenitor cells, cardiomyocytes or endothelial cells. Adding cardiac TCs might help promote cardiac regeneration and renewal.

## Introduction

Exercise has been proposed as an extremely effective and cheap intervention for the prevention and treatment of cardiovascular diseases 1. Healthy adults are recommended to conduct a moderate‐intensity exercise training for over 30 min./day and 5 days/week by the American Heart Association and the American College of Cardiology 2, 3, 4. Exercise can induce physiological cardiac growth, which is featured by enlarged cardiomyocyte cell size and formation of new cardiomyocytes 5. These newly formed cardiomyocytes could be either from pre‐existing cardiomyocytes or from cardiac progenitor/stem cells, which needs to be further clarified by myocyte‐restricted lineage tracing experiments 5. Exercise has been reported to activate cardiac stem cells while mammalian heart renewal has been supposed to be mainly mediated by pre‐existing cardiomyocytes 6, 7, 8, 9. Despite the unravelled cell source of these newly formed cardiomyocytes, understanding the molecular and cellular mechanisms of exercise‐induced cardiac growth might provide novel strategies to enhance the limited endogenous capacity for cardiac regeneration 2.

Several signalling pathways, including the IGF‐1‐PI3K‐Akt, nitric oxide, Eya2 and C/enhancer‐binding protein (EBP)β‐Cited4, have been identified to participate in exercise‐induced cardiac growth 5, 10, 11. Recently, miR‐222 has also been reported to be required for exercise‐induced cardiac growth 12. Actually, most of these studies focused on the effects of exercise on cardiomyocytes, however, exercise stimuli for healthy cardiac growth also affects other types of cardiac cells including telocytes (TCs), which is less well defined 2, 11, 12.

TCs are a recently identified distinct interstitial cell type, existing in many tissues and organs, including heart 13, 14, 15, 16. TCs can form a three‐dimensional (3D) interstitial network with neighbouring cells to maintain tissue homoeostasis by their extremely long and thin processes extended from the cell body namely telopodes 17, 18. Meanwhile, TCs contribute to intercellular communication by releasing small molecules or shed vesicles 19. Increasing evidence has demonstrated the existence of TCs in stem cells niches in different tissues and organs including cardiac stem cell niches, indicating their potential roles in regenerative medicine 20, 21. In heart, exercise can activate cardiac stem cells and induce formation of new cardiomyocytes 5, 6, 7, 8, 12. However, the response of cardiac TCs to exercise is still unclear.

In this study, taking advantage of the double immunofluorescence labelling for CD34 and vimentin, or platelet‐derived growth factor (PDGF) receptor‐α or ‐β, we found that in exercise‐induced cardiac growth, cardiac TCs were significantly increased, indicating that the increase in TCs might lead to the healthy heart growth in response to exercise and also help promote the beneficial cardiac phenotype in athletes.

## Materials and methods

### Mice and exercise

Male C57BL/6 mice aged 8–10 weeks, purchased from Shanghai Laboratory Animal Center (SLAC, Shanghai, China), were used in this study. All mice were allowed free access to food and tap water. This study was approved by the ethical committees of Shanghai University and all animal experiments were conducted under the guidelines on human use and care of laboratory animals for biomedical research published by National Institutes of Health (No. 85‐23, revised 1996).

Male mice swam in a ramp protocol starting at 10 min. twice a day. The increase in swimming time is 10 min./day until training time reached 90 min. twice daily. The entire exercise protocol was ended after 4 weeks. To avoid potential hypoxia and drowning under the water surface, these mice were closely observed all the time during the swimming exercise. Mice were intraperitoneally injected with 50 mg/kg of EDU (Invitrogen, Carlsbad, CA, USA) 48 hrs before sacrifice. At the end of 4 weeks, mice were sacked and the body weight, heart weight and tibia length were determined and compared with age‐matched male sedentary controls.

### RNA extraction and quantitative real‐time polymerase chain reactions

Total RNA was extracted from ventricle samples of mice using RNeasy Mini Kit (Qiagen, Hilden, Germany) according to the manufacturer's instructions. Through Bio‐Rad iScript^™^ cDNA Synthesis Kit (Bio‐Rad, Richmond, CA, USA), 500 ng of total RNA was reverse transcribed into cDNA. The RT product was subjected to 40 cycles of quantitative PCR with Takara SYBR Premix Ex Taq^™^ (Tli RNaseH Plus; Takara, Shiga, Japan) in CFX96^™^ Real‐Time PCR Detection System (Bio‐Rad). The primers used were as follows. 18S: 5′‐TCA AGA ACG AAA GTC GG AGG‐3′ and 5′‐GGA CAT CTA AGG GCA TCA C‐3′; ANP: 5′‐TGA CAG GAT TGG AGC CCA GAG‐3′ and 5′‐AGC TGC GTG ACA CAC CAC AAG‐3′; BNP: 5′‐GCT GCT TTG GGC ACA AGA TAG‐3′ and 5′‐GGT CTT CCT ACAACA ACT TCA‐3′. Relative mRNA expression levels were normalized to 18S by using 2^−ΔΔCt^ method.

### Immunofluorescent stainings

Ventricular tissues were snap frozen in optimal cutting temperature compound and were sectioned at 10 μm. First, sections were fixed in 4% paraformaldehyde followed by three times washes of PBS. Next, sections were incubated with 0.05% Triton‐X‐100 for 20 min. to penetrate the cytomembrane. After three time washes of PBS, sections were pre‐incubated with 4% bovine serum albumin solubilized in PBS for 1 hr.

For cell size staining, sections were incubated with Wheat Germ Lectin (WGA, 1:200; Sigma‐Aldrich, St. Louis, MO, USA) and DAPI (4′,6‐diamidino‐2‐phenylindole, 1:500; Life Technology, Lafayette, CO, USA) for 15 min. at room temperature. For EdU staining, sections were incubated overnight at 4°C with mouse monoclonal anti‐α‐actinin (1:200; Sigma‐Aldrich) and then exposed for 2 hrs to goat anti‐mouse labelled with Cy3 (1:200; Jackson, West Grove, PA, USA). After that, EdU (Invitrogen) was incubated for 30 min. in darkness at room temperature. Finally, sections were stained with DAPI (Life Technology).

For TCs staining, sections were incubated overnight at 4°C with rabbit monoclonal anti‐CD34 (1:200; Abcam, Cambridge, UK) and mouse polyclonal anti‐PDGF receptor‐α (1:200; Epitomics, Cambridge, UK), and then exposed for 2 hrs to goat anti‐mouse labelled with Cy3 (1:200; Jackson) and donkey anti‐rabbit labelled with fluorescein isothiocyanate (FITC) secondary antibodies (1:200; Jackson). Finally, sections were stained with DAPI (Life Technology). The same protocol was used for double immunofluorescent stainings for CD34/PDGF receptor‐beta (mouse monoclonal anti‐PDGFR‐β, 1:200; Epitomics) or CD34/Vimentin (mouse monoclonal anti‐Vimentin, 1:200; Epitomics).

At least 20 images (400×) were randomly chosen in the central area of each sections by using confocal laser scanning microscope (LSM 710; Carl Zeiss MicroImaging GmbH, Jena, Germany) and double immunofluorescent staining was merged by Zen 2011 software (Carl Zeiss MicroImaging GmbH). Counting was performed by two independent observers blinded to the sample classification. The density of TCs was expressed as average TC number in these 20 images.

### Statistical analysis

All data were presented as mean ± S.E.M. and statistically analysed by an independent Student's *t*‐test using SPSS 19.0 software (IBM, Armonk, NY, USA). A *P*‐value less than 0.05 was considered as statistically significant.

## Results

**Figure 1:**
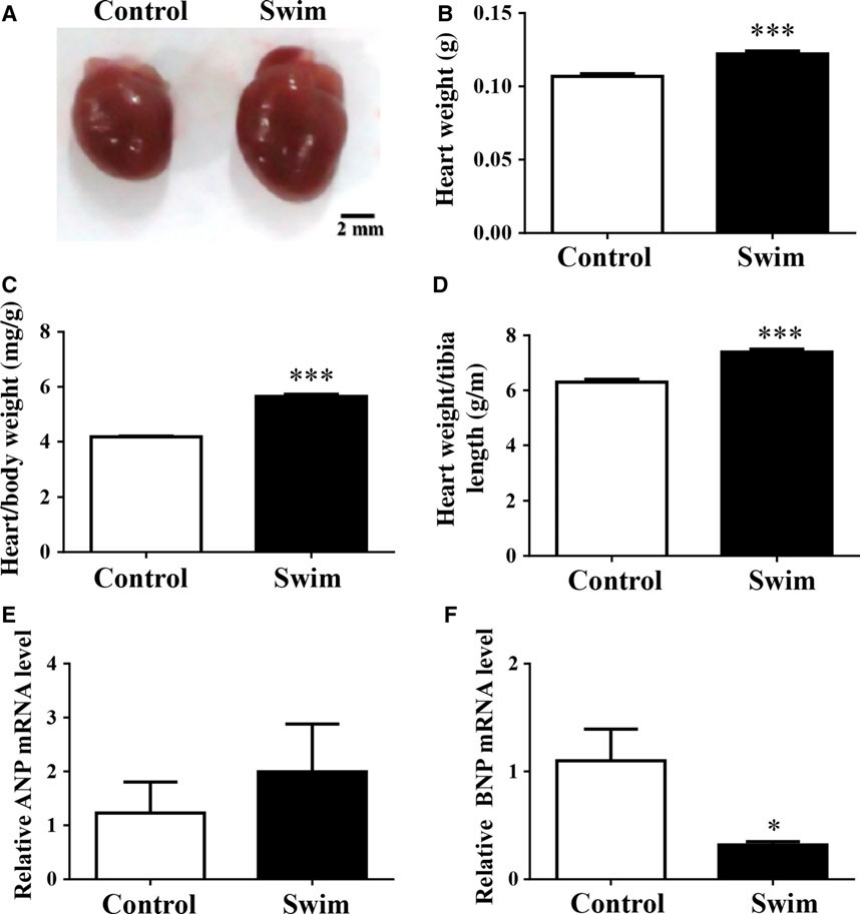
Exercise induces healthy cardiac growth. Representative images of general view of the heart (**A**), heart weight (**B**), heart weight/body weight ratio (**C**), heart weight/tibia length ratio (**D**), and relative ANP (**E**) and BNP (**F**) mRNA levels demonstrated that healthy cardiac growth was established after swimming training, scale bar = 2 mm. *n* = 5 for **A**–**C** while *n* = 4 for **E** and **F**. Compared to controls, *P < 0.05, ***P < 0.001.

A ramp swimming exercise murine model was used to investigate the response of cardiac TCs to exercise‐induced cardiac growth. After 4 weeks of swimming training, robust cardiac hypertrophy was induced as indicated by increased ratios of heart weight to body weight and heart weight to tibia length (Fig. 1A–D). Moreover, the expression levels of pathological cardiac hypertrophy markers including ANP and BNP were not elevated (Fig. 1E and F), confirming that a healthy cardiac hypertrophy model was established after swimming training. Therefore, exercise‐induced physiological cardiac growth was established in these mice.

**Figure 2:**
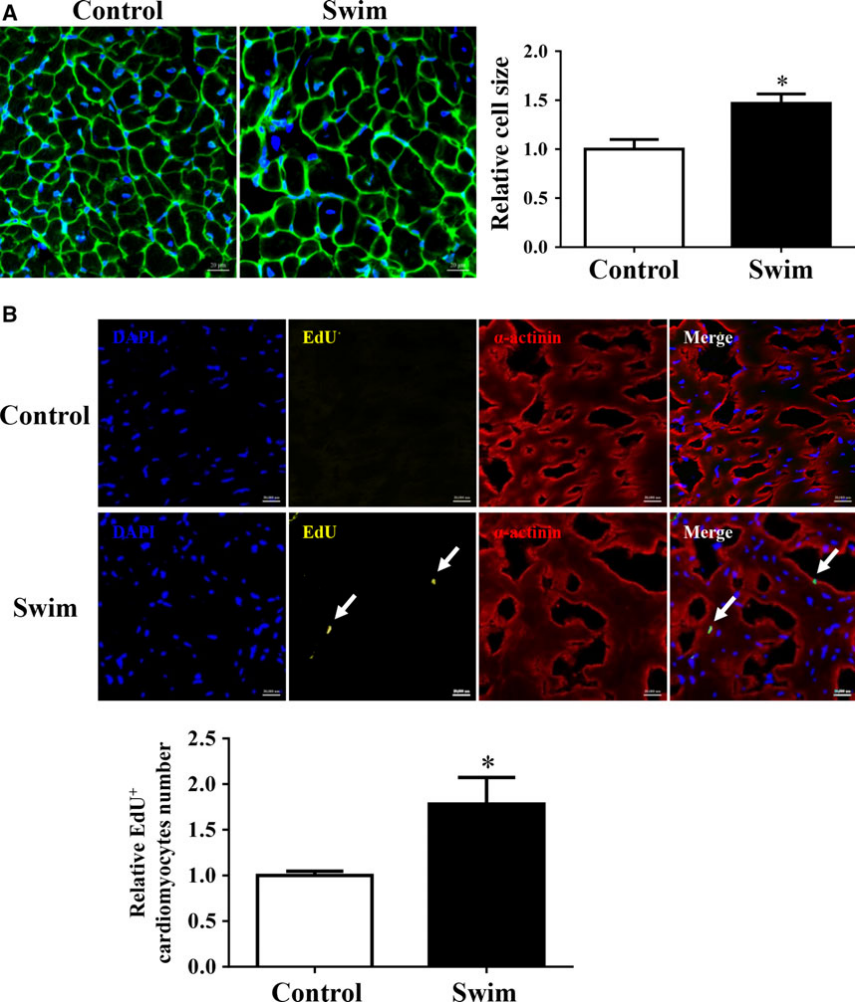
Exercise increases cardiomyocyte cell size and formation of new cardiomyocytes. Exercise increases cardiomyocyte cell size as determined by Wheat Germ Lectin (WGA) staining (**A**) and induces formation of new cardiomyocytes as determined by EdU staining (**B**), scale bar = 20 μm, *n* = 5. Compared to controls, *P < 0.05.

The main features of exercise‐induced physiological hypertrophy include increase in cardiomyocyte cell size and formation of new cardiomyocytes 5, 12. Here based on WGA staining, we confirmed that exercise can induce a 46% increase in cardiomyocyte cell size (Fig. 2A). Besides that, the concept that exercise can induce formation of new cardiomyocytes is promising as it might offer physiological cues that promote the limited endogenous capacity of the adult heart for regeneration 5, 12. In this study, we also demonstrated that exercise could induce formation of new cardiomyocytes based on EdU and α‐actinin double staining (Fig. 2B), a similar pattern that has been reported in previous studies 5, 12. Considering the potential role of TCs in tissue regeneration, it would be interesting to determine the response of cardiac TCs to exercise in these samples.

**Figure 3:**
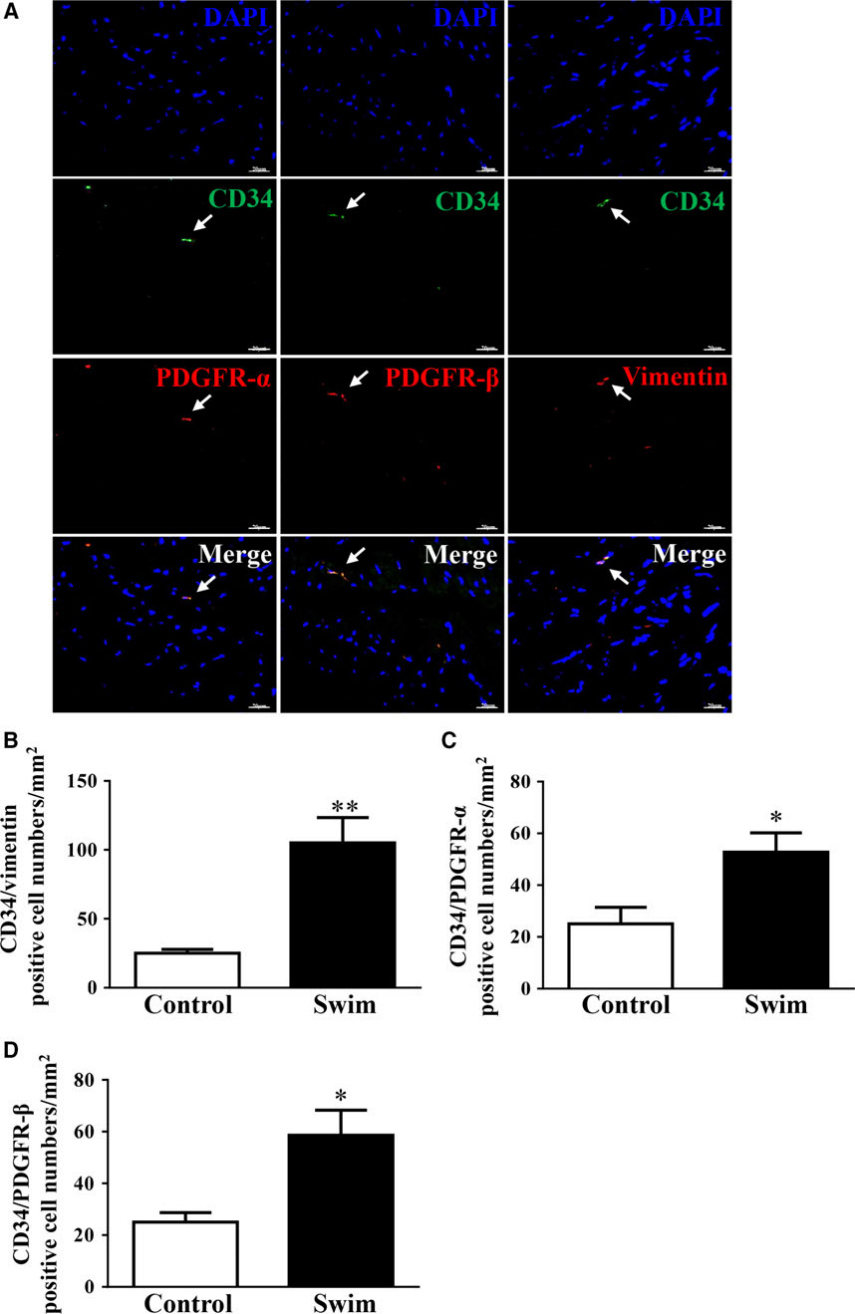
Telocytes (TCs) are increased in exercised heart. (**A**) Representative images of double immunostainings for cardiac TCs as determined by CD34/Vimentin, CD34/PDGFR‐α, and CD34/PDGFR‐β. Cardiac TCs are increased in response to exercise as determined by double immunofluorescence labelling for CD34/Vimentin (**B**), CD34/PDGFR‐α (**C**), and CD34/PDGFR‐β (**D**), scale bar = 20 μm, *n* = 5. Compared to controls, *P < 0.05, **P < 0.01.

Double‐immunostaining is currently the most common tool for semi‐quantitative analysis of TCs 15, 17, 22, which can also discriminate TCs from other types of interstitial cells though transmission electron microscopy (TEM) examination is a golden standard for TC identification 13, 17, 21. In this study, we used three distinct double‐immunostaining methods including double labelling for CD34/Vimentin, CD34/PDGFR‐α and CD34/PDGFR‐β to determine the changes in TC number in exercised heart (Fig. 3A). CD34 and Vimentin double labelling quantitative analysis indicated a significant increase in the number of TCs in exercised heart (Fig. 3B). Similarly, we also found that the number of cardiac TCs was increased in exercise‐induced cardiac growth as determined by CD34/PDGFR‐α and CD34/PDGFR‐β double‐immunostaining (Fig. 3C and D).

## Discussion

This study provides a novel finding that cardiac TCs are increased in exercise‐induced cardiac growth, similar to the recent reports that TCs are increased in partial hepatectomy‐induced liver regeneration and also in pregnancy‐induced physiological liver growth 20, 22. The data we presented here show that cardaic TCs might contribute to exercise‐induced cardiac growth.

The heart has a limited capacity to form new cardiomyocytes from pre‐existing cardiomyocytes or cardiac progenitor/stem cells 23. It has been demonstrated that modest cardiomyocyte turnover occurs in adult human or mouse hearts by pre‐existing cardiomyocyte proliferation 9, 24, 25. A recent study has further suggested a rare population of adult cardiomyocytes undergoing cell‐cycle entry and expansion in mouse heart by taking advantage of a new hypoxia‐induced fate mapping system 26. Exercise has been reported to be a stimulus for formation of new cardiomyocytes 5, 12. A proportionate growth of cardiomyocytes and non‐cardiomyocytes has been identified as characteristic of exercise‐induced physiological growth 10. TCs are a distinct interstitial cell type, with special cell morphologies and immunophenotypes, gene profiles, microRNA signatures, proteomic expressions and electrophysiological properties 27, 28, 29, 30, 31, 32, 33, 34, 35. Cardiac TCs have been proposed to form a tandem with cardiac stem/progenitor cells in cardiac stem cell niches, participating in cardiac regeneration and renewal 36, 37, 38. Based on three distinct double‐immunostainings for CD34/Vimentin, CD34/PDGFR‐α and CD34/PDGFR‐β, our data presented here consistently confirmed that cardiac TCs were increased in response to exercise, indicating a potential role of cardiac TCs in exercise‐induced cardiac growth. It would be interesting to determine the ultrastructural features of cardaic TCs in exercised heart by TEM or 3D imaging using focused ion beam scanning electron microscopy, and this should be noticed as a limitation of this study 37, 39, 40.

It remains to be checked if the increase in cardiac TCs in exercise helps forward the process of healthy cardiac growth or is just a consequence of that. Nevertheless, several potential roles of cardiac TCs could be herein proposed. First, the increase in cardiac TCs could contribute to the effects of exercise in promoting newly formed cardiomyocytes. TCs have been regarded as important interstitial cells to ‘nurse’ or ‘guide’ putative cardiomyocyte precursors to differentiate and integrate into heart architecture 17, 21, 38. TCs also communicate with cardiomyocytes either by direct contacts or shed vesicles, which could indeed probably affect pre‐existing cardiomyocyte cell fate in response to exercise. Interestingly, decreased numbers of cardiac TCs have been reported in myocardial infarction model and failing human heart 41, 42, 43, while intramyocardial transplantation of cardiac TCs could attenuate myocardial infarction and improve post‐infarcted cardiac function 44. Thus, the increase in cardiac TCs in exercise might also potentially contribute to cardiac regeneration and renewal under pathological conditions. Second, the increase in cardiac TCs in exercise may also participate in the maintenance of normal 3D‐organization of the extracellular matrix and balanced angiogenesis during cardiac growth, as TCs also form cellular junctions with other cell types such as fibroblasts, endothelial cells, pericytes and other interstitial cells in heart or interact with these cells *via* paracrine secretion including microRNAs, microvesicles or exosomes 36, 38, 45, 46. By this, TCs might be able to affect the activities of cardiac stem/progenitor cells, cardiomyocytes, endothelial cells or other interstitial cells during exercise‐induced cardiac growth 36, 43, 47, 48, 49.

In conclusion, this study shows the increase in cardiac TCs in exercise‐induced cardiac growth based on three distinct double immunoreactions. Adding cardiac TCs might help promote cardiac regeneration and renewal.

## Conflicts of interest

The authors declare there are no conflicts of interest.
